# Academic achievement and healthy lifestyle habits in primary school children: an interventional study

**DOI:** 10.3389/fpsyg.2024.1412266

**Published:** 2024-07-22

**Authors:** Maria Chiara Gallotta, Valerio Bonavolontà, Giovanna Zimatore, Davide Curzi, Lavinia Falcioni, Silvia Migliaccio, Laura Guidetti, Carlo Baldari

**Affiliations:** ^1^Department of Physiology and Pharmacology “Vittorio Erspamer”, Sapienza University of Rome, Rome, Italy; ^2^Department of Biotechnological and Applied Clinical Sciences, University of L’Aquila, L’Aquila, Italy; ^3^Department of Theoretical and Applied Sciences, eCampus University, Novedrate (CO), Italy; ^4^Niccolò Cusano University, Rome, Italy; ^5^Department of Health and Exercise Science, Appalachian State University, Boone, NC, United States; ^6^Department of Experimental Medicine, Sapienza University of Rome, Rome, Italy

**Keywords:** attentional performance, eating habits, fitness level, motor coordination, physical education intervention, weight status

## Abstract

**Purposes:**

First, to examine the relationship between primary school children’s academic achievement and healthy lifestyle habits. Second, to evaluate the effectiveness of two different 5-month physical education interventions (traditional physical education vs. coordinative physical education) on children’s academic achievement. Third, to examine whether variations of anthropometric variables, fitness level, gross motor coordination, physical activity level, sedentary time, attentional performance, fruit and vegetable consumption, meal frequency and type of physical education intervention could predict children’s academic achievement variations.

**Methods:**

Before and after the intervention, Italian language and mathematics skills, anthropometric variables (weight, height, body fat percentage, BMI), physical fitness (aerobic fitness, muscular strength, flexibility), gross motor coordination, attentional performance (processing speed, concentration performance, performance accuracy, attentional and inhibitory control), physical activity level, sedentary time and eating habits (meal frequency, fruit and vegetable consumption) were assessed in 161 Italian primary school children, randomly assigned to a traditional physical education group or to a coordinative physical education group.

**Results:**

Physical activity level, gross motor coordination and aerobic fitness moderately predicted mathematics skill (R^2^ = 17%). Moreover, physical activity level, aerobic fitness and muscular strength moderately predicted Italian language skill (R^2^ = 21%). Intervention type differently affected academic achievement. Specifically, Italian language and mathematics skills significantly improved only after traditional intervention. Fruit consumption increase was positively associated with the improvement in academic achievement. Coordinative physical education intervention was associated with a lower probability of improvement in Italian language and mathematical skills.

**Conclusion:**

Motor ability and lifestyle habits may have a positive influence on academic achievement in children. Unexpectedly, traditional physical education intervention resulted to be more effective on both Italian language and mathematical skills.

## 1 Introduction

Regular participation in physical activity among youth is positively associated with physical and psychological health and with some aspects of cognitive and academic performance (Bull et al., 2020). Physical activity could impact on academic performance through different direct and indirect learning, cognitive, emotional and physiological mechanisms (Rasberry et al., 2011; Barbosa et al., 2020). It was well demonstrated the close relationship between physical activity and academic achievement, behavior outcomes, psychosocial and cognitive functioning (Donnelly et al., 2016; Barbosa et al., 2020; Vasilopoulos and Ellefson, 2021). Moreover, many studies revealed the positive association between aerobic fitness, flexibility, and muscular strength (Gil-Espinosa et al., 2020) and the negative association between obesity (Martin et al., 2017) and sedentary time (Haapala et al., 2017) with academic achievement. Although research remains limited in investigating the relationship between healthy lifestyle habits (including physical activity, diet, screen time) and academic performance (García-Hermoso and Marina, 2017; Faught et al., 2017a) several studies showed consistent positive associations between fruit and vegetable consumption (Florence et al., 2008; Stea and Torstveit, 2014) and regular meal pattern (Stea and Torstveit, 2014) with better academic performance. In spite of these evidences, many children and adolescents have unhealthy eating habits, are engaged in more sedentary time than recommended, and do not meet the recommended daily 60 min of moderate-to-vigorous physical activity (Katzmarzyk et al., 2018; Bull et al., 2020; Paduano et al., 2021). In this context, school could have a crucial role to obtain changes on eating habits, physical activity level and sedentary behaviors in children. School-based food and nutrition education programs could promote the appropriate consumption of food in children since they usually consume one/two meals per day in school (Khambalia et al., 2012). School-based physical activity interventions could provide children the opportunity to participate in physical activity programs aimed at increasing physical activity level and promoting an active lifestyle (Gallotta et al., 2016, 2017). Moreover, school-based physical activity interventions could be useful to increase children’s physical fitness and motor coordination (Gallotta et al., 2017), cognitive development (Gallotta et al., 2015; García-Hermoso et al., 2021) and academic performance (García-Hermoso et al., 2021; Loturco et al., 2022).

To date, cross-sectional (Faught et al., 2017a; Bleiweiss-Sande et al., 2019) and interventional studies (Wassenaar et al., 2020; García-Hermoso et al., 2021; Takehara et al., 2021) examined the relationship between some of these factors and academic achievement in children and adolescents. These studies also showed the need to better understand the relationships between multiple lifestyle factors and academic achievement.

Moreover, the long-term effects of physical exercise on children’s academic performances have been studied by previous authors after manipulating quantitative aspects of physical exercise (Donnelly et al., 2016; Bugge et al., 2017; García-Hermoso et al., 2021). To our knowledge, no data are available regarding the effects of the manipulation of qualitative aspects of physical activity on children’s academic achievement. The quality of physical activity in children primarily concerns the variety and the level of coordinative demands of the physical exercise contents (Gallotta et al., 2009). It is focused on the manipulation of the complexity of the physical exercise tasks thus requiring mental engagement (Tomporowski et al., 2015). Thus, physical education may provide children with a high-quality experience of physical activity, enhancing cognitive functions such as memory, reaction speed, attention, and concentration. This, in turn, can facilitate the learning process and lead to improved academic performance.

Previous studies demonstrated that long-term coordinative exercises had positive effects on children’s cognition (Gallotta et al., 2015; Tomporowski et al., 2015) and that children’s motor coordination was positively associated with their academic achievement (Ryu et al., 2021). Coordinative exercises include complex movements that place demands on children’s executive processes (Best, 2010; Gallotta et al., 2015), providing a constructive basis for improved cognitive performances and therefore for the academic performances that are closely linked with them (Gunzenhauser and Nückles, 2021). The most available cross-sectional studies (Stea and Torstveit, 2014; Donnelly et al., 2016; García-Hermoso and Marina, 2017; Faught et al., 2017a; Barbosa et al., 2020) also showed the need to conduct longitudinal interventional designs to establish the causal direction of the association between multiple lifestyle factors and academic achievement.

Therefore, the first aim of this study was to examine the association between academic achievement (Italian language and mathematics skills) and anthropometric variables, fitness level (aerobic fitness, muscular strength, flexibility), gross motor coordination, physical activity level, sedentary time, attentional performance, fruit and vegetable consumption and meal frequency in primary school children.

The second aim of this study was to evaluate the effect of a qualitative physical education intervention on children’s academic achievement by proposing two different exercise programs: a traditional physical education intervention and a coordinative physical education intervention conducted by a specialist physical education teacher. We hypothesized a possible selective improvement of children’s academic performance in the coordinative group compared to the traditional group.

The third aim of this study was to examine whether variations of anthropometric variables, fitness level (aerobic fitness, muscular strength, flexibility), gross motor coordination, physical activity level, sedentary time, attentional performance, fruit and vegetable consumption, meal frequency and type of physical education intervention could predict children’s academic achievement variations (Italian language and mathematics skills).

## 2 Materials and methods

### 2.1 Selection of schools and allocation to intervention

This intervention study was conducted in all classes (from Grade 3 to Grade 5) of three primary schools in a rural area close to Rome (Italy). The area was included in a circle of 5 km radius from a landmark to obtain a sample with comparable environmental characteristics. This comprised a total of 5 schools. We further excluded those schools that were already engaged in physical activity programmes / interventions. The remaining 3 schools were invited and agreed to participate in the study. The schools were the experimental units that received the intervention and, therefore, were either randomized to traditional physical education group or coordinative physical education group (Thomas et al., 2015).

### 2.2 Participants

Among the 266 eligible children, one hundred and sixty-one primary school children aged 8–11 years volunteered to participate in this study. The distribution of students in the classroom was as follows: there were 55 Grade 3 children aged 8 to 9 years, 62 Grade 4 children aged 9 to 10 years, and 44 Grade 5 children aged 10 to 11 years. After the randomization process, the traditional physical education group consisted of 78 participants (33 girls and 45 boys), while the coordinative physical education group comprised 83 participants (38 girls and 45 boys). Children were eligible if they had no attention-deficit disorders, academic and learning difficulties, dyslexia, developmental and neurological disorders, medical conditions that would affect study results or limit physical activity. All children met the inclusion criteria.

The University Ethical Committee approved this investigation (Rif 3,502 Prot. 1883/15) in accordance with the ethical standards laid down in the 1964 Declaration of Helsinki and its later amendments.

Informed written consent was obtained from both parents prior to study participation.

### 2.3 Variables assessment

#### 2.3.1 Academic achievement

Before and after the intervention, children’s academic achievement (Italian language and mathematics skills) was rated made by generalist school teachers on a 5-point rating scale ranging from 1 to 5 with ascending numbers indicating higher achievement (1: much below average, 2: below average, 3: average, 4: above average, 5: much above average) (Henricsson and Rydell, 2006; Thorell et al., 2013).

#### 2.3.2 Anthropometric variables

Before and after the intervention, children’s weight, height, body mass index (BMI), and body fat mass percentage (FM%) were evaluated. Weight and height measurements were taken using a scale and a stadiometer, respectively, with accuracy to the nearest 0.5 kg and 0.1 cm. BMI was calculated by dividing the weight in kilograms by the square of the height in meters. The FM% was determined through a multi-frequency hand-to-foot bioelectrical impedance method (IOI 353 analyzer, Jawon Medical Co. Ltd., Seoul, South Korea).

#### 2.3.3 Physical fitness assessment

##### 2.3.3.1 Aerobic fitness

Before and after the intervention, children’s aerobic fitness level was evaluated using the PACER test. Children had to run as long as possible back and forth across a 15-meter space at a specified pace that got faster each minute (Welk et al., 2013). Then, a conversion chart was used to convert scores on the 15-M PACER to a 20-M score (Welk et al., 2013) to enter the Léger equation (Léger et al., 1988) for estimating VO_2max_ values (ml. kg^−1^. min^−1^).

##### 2.3.3.2 Muscular strength and endurance

Before and after the intervention, children’s muscular strength was assessed by the curl-up and push-up tests for abdominal muscles and upper body strength and endurance, respectively. Children had to perform as many curl-ups as possible until reaching a maximum of 75 repetitions and as many 90° push-ups as possible at a specified pace. The score was the number of correctly performed curl-ups and push-ups (Welk et al., 2013).

From the two tests, a single muscular strength z-score was calculated. Each individual test score was standardized using the formula: z-standardized value = (value − mean)/SD. The muscular strength z-score was obtained by averaging the two standardized scores (Martinez-Gomez et al., 2012).

##### 2.3.3.3 Flexibility

Before and after the intervention, children’s flexibility was assessed by the back-saver sit and reach test. Children had to reach as far as possible with one leg straight while sitting at a sit-and-reach box. The measurement was performed on one side (right, left) at a time. The score was recorded to the last whole centimeter, with distances above 30 cm being recorded as 30 (Welk et al., 2013).

#### 2.3.4 Gross motor coordination

Before and after the intervention, children’s gross motor coordination was assessed by the four subtests (balance beam test, jumping laterally test, hopping on one leg over an obstacle test, shifting platforms test) included in the Körperkoordinationstest Für Kinder battery (Kiphard and Schilling, 2017). The motor quotient (MQ), a comprehensive indicator of motor coordination adjusted for age and gender, was then computed using the raw values obtained from the four subtests included in the battery (Kiphard and Schilling, 2017).

#### 2.3.5 Attentional performance

Before and after the intervention, children’s attentional performance was evaluated using the d2-R test of attention. This test aimed to assess their ability to concentrate on a specific stimulus or task while disregarding distractions from competing stimuli. The d2-R test is a paper-and-pencil letter cancellation test to assess concentration and sustained attention under stress induced by time constraints. Children’s processing speed and amount of work completed (TN), concentration performance (CP), performance accuracy (E%), and attentional and inhibitory control (TP) were assessed (Brickenkamp et al., 2013).

#### 2.3.6 Physical activity level

Before and after the intervention, children’s physical activity level was evaluated using the self-administered Italian version of the Physical Activity Questionnaire for Older Children (PAQ-C-It) (Gobbi et al., 2016). The questionnaire is a 7-day recall instrument comprising nine questions related to sports, games, physical activities at school, and leisure-time activities, including the weekend. Each question is rated on a scale from 1 to 5, and the final score is calculated by averaging the scores from all the questions (Crocker et al., 1997).

#### 2.3.7 Sedentary time

Before and after the intervention, self-reported sedentary time was evaluated through a parental proxy interview. Children’s parents were requested to provide information on the average number of minutes their children spent reading, watching television, playing video games, and using the computer on both weekdays and weekends, excluding school hours (Coombs et al., 2013).

#### 2.3.8 Fruit and vegetable consumption

Before and after the intervention, children’s fruit and vegetable consumption was assessed by a 7-day diet record. Children were asked about their weekly fruit and vegetable consumption frequency, with response options scored as follows: 1 = “never,” 2 = “less than once a week,” 3 = “once a week,” 4 = “2 to 4 days a week,” 5 = “5 to 6 days a week,” 6 = “once a day, every day,” and 7 = “every day, more than once” (Vereecken et al., 2008).

#### 2.3.9 Meal frequency

Before and after the intervention, children’s meal frequency was assessed by a 7-day recall questionnaire. Meal frequency was assessed by questions such as: “How many times do you have breakfast in a week?” The same was asked for lunch, dinner, mid-morning and afternoon meals. Response alternatives ranged from never to 7 days a week. The response options for all items were scored as 1 = “never/once a week,” 2 = “2 to 3 times a week,” 3 = “4 to 6 times a week,” 4 = “every day” (Nardone et al., 2015).

Prior to the administration, children were given instructions to complete all the questionnaires, which were group-administered in classrooms under quiet conditions. They were provided with sufficient time to fill out the questionnaires, and an experimenter was present to address any questions raised by the children.

### 2.4 Physical education intervention

Both physical education interventions were conducted over a period of 5 months, comprising two 1-h sessions per week. The interventions were designed and led by the same specialist physical education teacher. The two interventions varied in the type and mode of physical activities in which children participated, but they were similar in terms of their overall structure, duration, and relative intensity. The OMNI scale (Utter et al., 2002) was used to monitor intensity and ensure that there were no differences between the two interventions. In both interventions, each lesson consisted of a 15-min warm-up, followed by 35 min of moderate-to-vigorous physical activities (MVPA) with an intensity level ranging from 5 to 8 RPE (U.S. Department of Health and Human Services, 2008). Finally, each session concluded with 10 min of cooldown and stretching exercises.

The *traditional* physical education intervention aimed primarily to enhance children’s flexibility, strength, and cardiovascular endurance development (DPR, 2013). The specialist physical education teacher proposed flexibility and strength exercises and circuit training for cardiovascular health without complex coordinative demands with the main goal of developing children’s fitness and health (Gallotta et al., 2015, 2016, 2017) (see Appendix for full protocol description).

The *coordinative* physical education intervention aimed primarily at fostering the development of children’s coordination and dexterity. It consisted of four didactic modules (sport-games module, rhythmic activities module, gymnastics module, and fitness activities module) designed to offer children diverse opportunities to engage in and learn various sports and unconventional activities.

The *sport-games module* was characterized by the sport-unspecific use of different types of balls in the context of mini-games.

The *rhythmic activities* module was characterized by the execution of rhythmic sequences on a musical base with small tools.

The *gymnastics module* was characterized by the high variety of preparatory exercises for basic gymnastics fundamentals.

The *fitness activities module* was characterized by exercises with high coordinative demands to favor strength, endurance, speed, and flexibility development (Gallotta et al., 2015, 2016, 2017) (see Appendix for full protocol description).

### 2.5 Statistical analysis

#### 2.5.1 General characteristics of the participants

Children’s baseline characteristics by intervention group (traditional physical education group / coordinative physical education group) were described by means and standard deviations.

#### 2.5.2 Association between academic achievement and all other measured variables

Pearson’s correlation analysis was used to explore the relationships between academic achievement (Italian language and mathematics skills) and anthropometric (BMI, %FM), aerobic fitness (VO_2max_), muscular strength (muscular strength z-score), flexibility, gross motor coordination (MQ), attentional (TN, CP, E%, TP), physical activity (physical activity level, sedentary time), fruit and vegetable consumption, and meal frequency variables before intervention. A multiple linear stepwise regression analysis was then performed to examine the associations of academic achievement with correlated variables at baseline.

#### 2.5.3 Evaluation of the effects of different physical education interventions on academic achievement

All results were expressed as mean ± standard deviation. Within the intervention type, differences in the baseline academic achievement scores were verified by means of an unpaired comparison t-test. Thus, academic achievement scores were analyzed using a 2 × 2 mixed ANOVA with intervention type (traditional vs. coordinative) as between factor and time (pre vs. post) as within factor. Effect size was also calculated using Cohen’s definition of small, medium, and large effect size (as partial ƞ^2^ = 0.01, 0.06, 0.14). Significant interactions were further analyzed by means of appropriate *post hoc* analysis.

For the academic achievement scores evaluated after the intervention, we calculated the absolute variation (∆) and the percentage of variation (∆%) with respect to its preintervention value (postintervention value ─ preintervention value). An unpaired comparison t-test was then performed to examine differences between the two types of intervention on ∆ and on ∆% in academic achievement scores.

#### 2.5.4 Relationship between academic achievement variations and several potential predictors

We calculated ∆ for all other variables. A multinomial logistic regression analysis was then used to assess whether the absolute variation of BMI, FM%, aerobic fitness level (VO_2max_), muscular strength (muscular strength z-score), flexibility, gross motor coordination (MQ), attentional performance (TN, CP, E%, TP), physical activity level, sedentary time, fruit and vegetable consumption, meal frequency and intervention type predicted academic achievement categories. The absolute variation of academic achievement was examined as three categories (improvement, invariance, and worsening). “Invariance category” was set as reference group. Intervention type was added as factor, the absolute variation of BMI, FM%, aerobic fitness level, muscular strength, flexibility, gross motor coordination, attentional performance, physical activity level, sedentary time, fruit and vegetable consumption and meal frequency were included in the analyses as covariates. All variables were tested in the same model, controlling the effect of each other.

Statistical significance was defined as *p* ≤ 0.05. Statistical analysis was performed with SPSS Version 27.0 statistic software package.

## 3 Results

Children’s baseline characteristics by intervention group are shown in Table 1.

**Table 1:** Children’s baseline characteristics of traditional physical education group and coordinative physical education group (mean ± standard deviation).

	Traditional PE group			Coordinative PE group		
	(*n* = 78)			(*n* = 83)		
Weight (kg)	38.8	±	11.5	37.3	±	8.9
Height (cm)	137.3	±	8.7	137.8	±	7.3
BMI (kg/m^2^)	20.2	±	4.1	19.5	±	4.0
FM%	20.3	±	8.6	19.2	±	8.6
Physical activity level (score)	2.2	±	0.7	2.2	±	0.7
Sedentary time (min)	678.5	±	232.3	478.6	±	251.8
TN (score)	370.6	±	81.4	404.7	±	95.7
CP (score)	128.8	±	32.3	103.3	±	41.3
E%	7.4	±	4.4	16.9	±	8.4
TP (score)	343.5	±	78.3	335.5	±	82.9
Italian language skill (score)	3.2	±	1.1	3.0	±	1.1
Mathematics skill (score)	3.1	±	1.1	3.2	±	1.2
Flexibility (cm)	16.2	±	5.6	15.9	±	6.2
Muscular strength (*z*-score)	0.0	±	0.8	0.2	±	0.7
VO_2max_ (ml·kg^−1^·min^−1^)	43.8	±	2.4	42.7	±	3.5
MQ (score)	76.2	±	12.1	77.1	±	11.6
Fruit consumption (score)	4.1	±	1.7	4.6	±	1.7
Vegetables consumption (score)	4.1	±	1.6	4.2	±	1.6
Breakfast meal frequency	3.7	±	0.7	3.6	±	0.8
Mid-morning meal frequency	2.6	±	0.5	2.6	±	0.5
Lunch meal frequency	2.9	±	0.3	2.8	±	0.5
Afternoon meal frequency	2.4	±	0.6	2.6	±	0.6
Dinner meal frequency	2.9	±	0.2	2.8	±	0.4

PE, physical education.

### 3.1 Association between academic achievement and all other measured variables

Aerobic fitness, muscular strength, gross motor coordination, physical activity level and dinner meal frequency at baseline significantly correlated with Italian language skill, while aerobic fitness, muscular strength, gross motor coordination and physical activity level at baseline significantly correlated with mathematics skill (Table 2).

**Table 2:** Correlation coefficients between academic achievement and variables measured at baseline.

	Italian language skill	Mathematics skill
Aerobic fitness (VO_2max_)	0.319^**^	0.277^**^
Muscular strength (*z*-score)	0.249^**^	0.221^**^
Gross motor coordination (MQ)	0.298^**^	0.294^**^
Physical activity level (score)	0.338^**^	0.309^**^
Dinner meal frequency	0.162^*^	

^**^p ≤ 0.01 ^*^p ≤ 0.05.

The application of the multiple regression analysis indicated that physical activity level, aerobic fitness and muscular strength at baseline predicted Italian language skill, although the percentage of variance it could explain was moderate (R^2^ = 21%) (Table 3).

**Table 3:** Multiple linear stepwise regression with Italian language skill as the dependent measure.

	Predictor variables	SE	Adjusted *R*^2^	*R* ^2^	Change in *R*^2^	*p*	*t*	β	F
Model 1	Physical activity level (score)	0.118	0.109	0.114	0.114	0.000	4.501	0.338	20.260
Model 2	Physical activity level (score)	0.116				0.000	3.907	0.288	
	Aerobic fitness (VO_2max_)	0.027	0.172	0.182	0.068	0.000	3.597	0.265	12.941
Model 3	Physical activity level (score)	0.116				0.000	3.589	0.263	
	Aerobic fitness (VO_2max_)	0.027				0.001	3.384	0.247	
	Muscular strength (*z*-score)	0.103	0.196	0.211	0.029	0.018	2.392	0.174	5.720

Variables used: aerobic fitness (VO_2max_). muscular strength (muscular strength z-score), gross motor coordination (MQ), physical activity level, dinner meal frequency at baseline.

Moreover, the multiple regression analysis indicated that physical activity level, gross motor coordination and aerobic fitness at baseline predicted mathematics skill, although the percentage of variance it could explain was moderate (R^2^ = 17%) (Table 4).

**Table 4:** Multiple linear stepwise regression with mathematics skill as the dependent measure.

	Predictor variables	SE	Adjusted *R*^2^	*R* ^2^	Change in *R*^2^	*p*	*t*	β	F
Model 1	Physical activity level (score)	0.120	0.089	0.095	0.095	0.000	4.077	0.309	16.625
Model 2	Physical activity level (score)	0.120				0.001	3.392	0.256	
	Gross motor coordination (MQ)	0.006	0.136	0.147	0.052	0.002	3.085	0.233	9.520
Model 3	Physical activity level (score)	0.119				0.002	3.137	0.236	
	Gross motor coordination (MQ)	0.007				0.023	2.303	0.181	
	Aerobic fitness (VO_2max_)	0.029	0.156	0.172	0.025	0.033	2.151	0.168	4.628

Variables used: aerobic fitness (VO_2max_), muscular strength (muscular strength z-score), gross motor coordination (MQ), physical activity level at baseline.

### 3.2 Evaluation of the effects of different physical education interventions on academic achievement

Differences in the baseline academic achievement scores of traditional group and coordinative group were verified (*p* < 0.05), but no significant differences were revealed.

**Figure 1:**
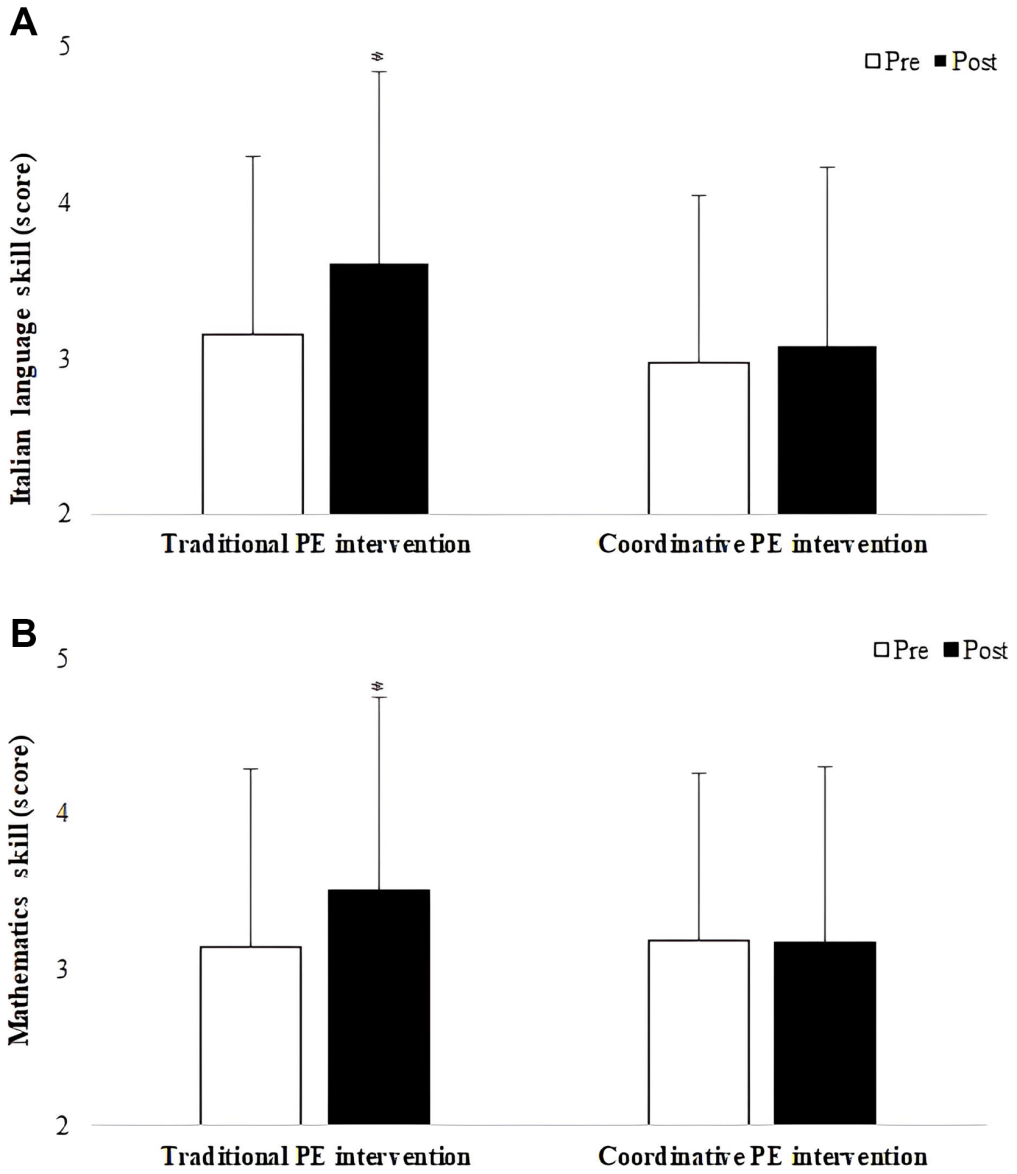
Pre- and post- intervention individual scores (mean ± SD) for Italian language **(A)** and mathematics **(B)** skills. Abbreviation: PE, physical education. *p < 0.001 post vs pre.

The main effect of time revealed that children’s Italian language (F_1,158_ = 32.73, *p* < 0.001, ƞ^2^ = 0.172) (3.06 ± 1.11 score vs. 3.34 ± 1.21 score) and mathematics (F_1,159_ = 16.05, p < 0.001, ƞ^2^ = 0.092) (3.17 ± 1.11 score vs. 3.34 ± 1.12 score) skills significantly improved after intervention. Moreover, ANOVA revealed a significant time x intervention type interaction on Italian language (F_1,158_ = 12.37, p < 0.001, ƞ^2^ = 0.073) and mathematics (F_1,159_ = 20.80, p < 0.001, ƞ^2^ = 0.116) skills, indicating the likely presence of differential effects of intervention type on academic achievement following the intervention. Specifically, Italian language and mathematics skills significantly improved only after traditional intervention (Figure 1).

**Figure 2:**
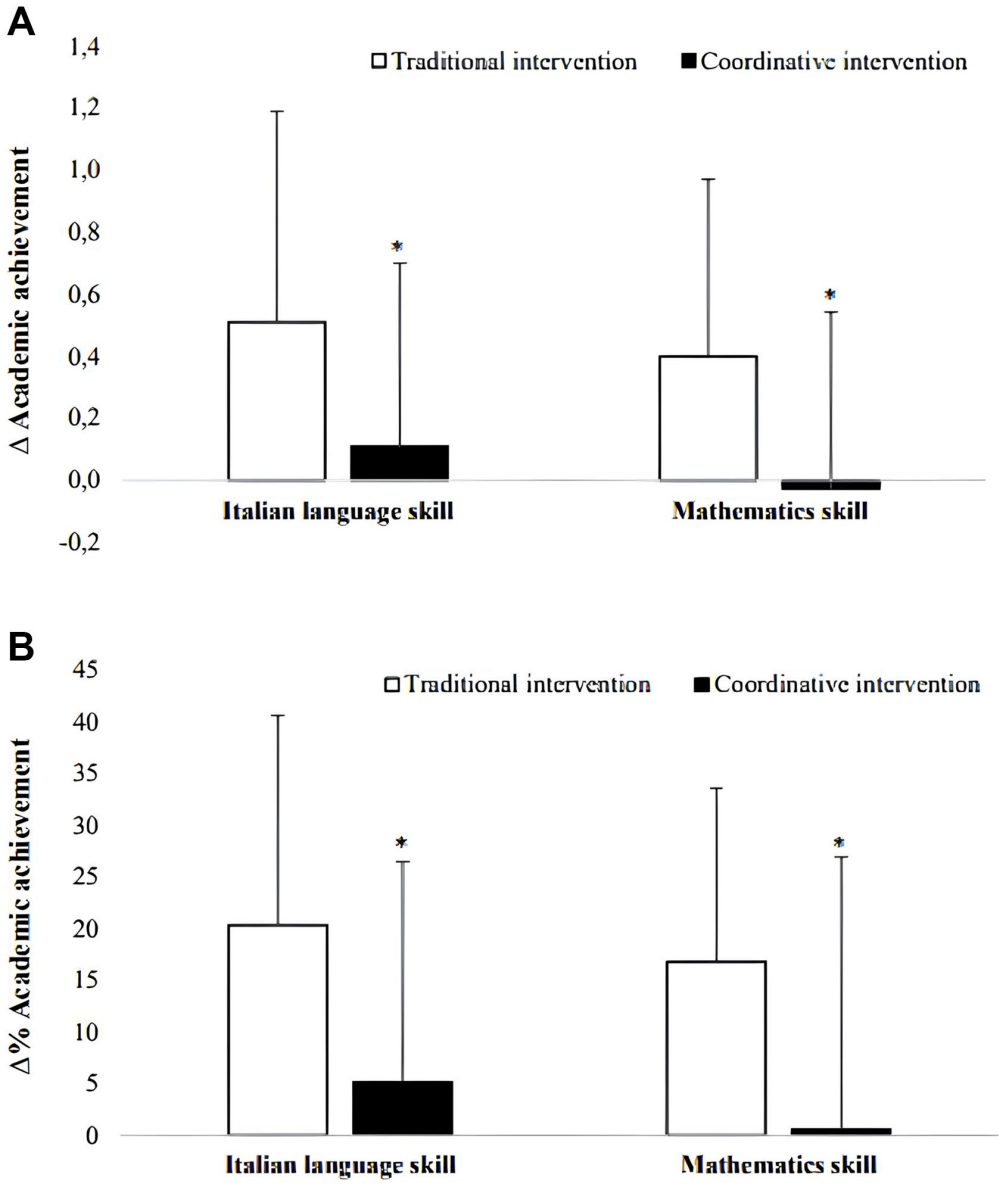
Variation (∆) **(A)** and parcentage of variation (∆%) **(B)** of academic achievement (± SD). *p < 0.001 traditional intervention vs coordinative intervention.

Improvements across the intervention were analyzed using ∆ and ∆%. The t-test comparison revealed that traditional intervention led to a higher improvement of academic achievement than coordinative intervention (Figure 2).

### 3.3 Relationship between academic achievement variations and several potential predictors

In order to model the relationship between the variation of academic achievement (academic achievement categories) and the variation of several potential predictors (BMI, FM%, aerobic fitness level, muscular strength, flexibility, gross motor coordination, attentional performance, physical activity level, sedentary time, fruit and vegetable consumption, meal frequency and intervention type) a multinomial logistic regression was performed. The variation of sedentary time [χ^2^ (2) = 10.05, *p* = 0.007], fruit consumption [χ^2^ (2) = 6.40, *p* = 0.04], and intervention type [χ^2^ (2) = 7.31, *p* = 0.03] contributed significantly to the variation of Italian language skill. The variation of afternoon meal frequency [χ^2^ (2) = 9.36, *p* = 0.009] and intervention type [χ^2^ (2) = 9.35, p = 0.009] contributed significantly to the variation of mathematics skill. Table 5 presents the results of the multinomial logistic regression.

**Table 5:** Multinomial logistic regression predicting academic achievement categories.

	**Improvement**	**Worsening**
Italian language skill	OR (95% CI)	OR (95% CI)
∆ sedentary time	1.00 (1.00–1.01)^**^	1.00 (1.00–1.00)
∆ fruit consumption	1.26 (1.03–1.53)^*^	0.93 (0.66–1.32)
Intervention type^a^		
Coordinative intervention	0.26 (0.09–0.75)^*^	1.22 (0.19–7.71)
Mathematics skill		
∆ afternoon meal frequency	3.30 (1.38–7.91)^**^	0.56 (0.14–2.30)
Intervention type^a^		
Coordinative intervention	0.26 (0.08–0.88)^*^	10.59 (0.52–215.19)

Invariance was chosen as reference group for the outcome. All variables were tested in the same model, controlling the effect of each other.

^*^p < 0.05, ^**^p < 0.01. OR odds ratio, CI confidence interval, CP concentration capacity.

a *Reference category is “traditional intervention”.*

Sedentary time and fruit consumption increases were positively associated with the improvement in Italian language skill. Moreover, coordinative physical education intervention was associated with a lower probability of improvement in Italian language skill. A lower variation (reduction) of afternoon meal frequency was positively associated with the improvement in mathematical skill. Moreover, coordinative physical education intervention was associated with a lower probability of improvement in mathematical skill.

## 4 Discussion

### 4.1 Association between academic achievement and all other measured variables

The first aim of the present study was to examine the association between a wide range of individual, physical, cognitive and nutritional variables and the academic achievement of primary school children.

Results revealed that aerobic fitness, muscular strength, gross motor coordination, physical activity level and dinner meal frequency were related to Italian language skill, while aerobic fitness, muscular strength, gross motor coordination and physical activity level positively correlated with mathematics skill. Additionally, results of the multiple regression analysis revealed that the level of physical activity and aerobic fitness were significant predictors of both Italian language and mathematics skills. Previous studies suggested that physical activity may impact on academic performance through a variety of direct and indirect emotional, cognitive, physiological, and learning mechanisms, revealing that cognitive and motor abilities develop concurrently (Rasberry et al., 2011). Children who have more and more well-developed motor abilities could attain a greater range of motor experiences that create the foundation for better academic performances (Salaj and Masnjak, 2022). The pre-frontal cortex brain area is involved in both the processing of motor information and cognitive tasks. Therefore, children with greater motor abilities show better achievement, since the stronger motor skills strengthen the neural connections that also assist children in many academic tasks (Tomporowski and Pesce, 2019). Moreover, evidence supported the relationship of physical activity and aerobic fitness with brain structure and function, and therefore with cognition and learning (Donnelly et al., 2016; Erickson et al., 2019). Physical activity causes changes to neural architecture and to brain function, inducing consequently changes in cognitive performance (Meijer et al., 2020). There is evidence suggesting that physical activity provides selective advantages to neural structures that specifically support certain aspects of cognition. Furthermore, research indicates that higher aerobic fitness might exert a targeted impact on cognitive functions, which are underpinned by specific brain structures. Research findings provide evidence that physical activity and physical fitness positively influence children’s mental functioning, indicating that children who are physically fit tend to outperform less-fit children on cognitive tasks. Physical activity-related changes in children’s brain function and cognition (e.g., concentration, attention, memory, information processing) induce gains in academic performance, also providing opportunities for fundamental motor skill acquisition (Donnelly et al., 2016; Erickson et al., 2019). Furthermore, it has been observed that physically fit children exhibit quicker and more robust neuro-electrical brain responses during reading compared to their less-fit peers. This finding suggests that a higher level of fitness may be linked to a more extensive network of words and their meanings, along with an improved capacity to identify and/or correct syntactic errors (Scudder et al., 2014).

### 4.2 Evaluation of the effects of different physical education interventions on academic achievement

The second aim of the present study was to evaluate the effect of a traditional physical education intervention and of a coordinative physical education intervention on children’s academic achievement. Our findings revealed a general improvement of academic skills after intervention, suggesting that physical activity may enhance children’s academic performance and cognitive outcomes (Donnelly et al., 2016; Barbosa et al., 2020; Vasilopoulos and Ellefson, 2021) and confirming that children engaged in regular physical exercise may perform much better in mathematics and language skills than inactive students (Álvarez-Bueno et al., 2017). Moreover, the positive effects of physical education interventions on academic performance could be due to the facilitating effects of physical activity on children’s mental function, intelligence and cognitive development (Singh et al., 2019).

Davis et al. (2011) proposed the existence of a direct path between exercise and cognitive performance due to a direct result of neural stimulation by movement. Currently, it is hypothesized that a positive effect of physical activity on cognitive functions is partly caused by physiological changes in the body such as increased levels of brain-derived neurotrophic factor (BDNF), that facilitates learning and maintains cognitive functions by improving synaptic plasticity and acting as a neuroprotective agent, increased brain circulation and improved neuroelectric functionality (Latomme et al., 2022). Therefore, neuroscientific evidence of the beneficial effects of exercise and physical activity on cognition supports a three-level model to explain the interaction between brain and movement: (i) increased vascularization augmenting brain activity; (ii) the release of neurotransmitters and BDNF which favor neurogenesis, memory, attention and motivation; (iii) the development of complex movement-related neural circuits and their interconnection with the executive brain functions (Doherty and Forés Miravalles, 2019). Moreover, different psychosocial mechanisms that accompany physical activity could induce the improvement of cognitive and academic performances after chronic exercise (Davis and Lambourne, 2009; Vella et al., 2023). In particular, participation in well-structured organized physical activity could positively impact on psychological variables such as mastery and self-efficacy, contributing to beneficial cognitive changes (Davis and Lambourne, 2009; Vella et al., 2023).

Engaging in structured and complex physical activities and games that involve motor learning, interaction with the environment, and group cooperation demand mental processes inherent to exercises that can enhance cognitive functions, including attention and concentration capacity. Thus, children engaged in physical activities that promote cooperation, sharing, and learning to follow rules learn skills that transfer to classroom setting (Tomporowski et al., 2008). Moreover, physical activity during the school day may induce arousal and reduce boredom, which can lead to increased attention span and concentration (Tomporowski et al., 2008).

However, our findings revealed that the type of intervention had differential effects on academic variables. In particular, results showed that the traditional physical education intervention led to greater improvement on both Italian language and mathematical skills, compared to the coordinative physical education intervention. The improvement of Italian language skills could be associated with structural and functional cortical development (Martin-Martinez et al., 2023) induced by the traditional physical education intervention which also included complex aerobic activities (e.g., running games) that increased activity in the prefrontal cortex and improved performance on tasks requiring executive functioning as well as having a positive effect on mathematics achievement (Davis et al., 2007). Moreover, the use of various number and counting movement games and activities during the traditional physical education intervention could justify the greater improvement on mathematical skills after this intervention.

Our results were not in line with previous studies suggesting that physical exercise involving greater coordinative and attentional demands had a great beneficial effect on cognitive performance (Tomporowski and Pesce, 2019; Kolovelonis et al., 2022), due to the well-documented close relationship between motor coordination and cognitive performance (Schmidt et al., 2017) and the activation of specific neuronal structures, such as the frontal cortex and the cerebellum, which are common to both cognition and motor coordination (de Bruijn et al., 2021).

We supposed that the lower improvement of children’s academic performance after the coordinative physical education intervention could be due to the excessive stress induced by the mixed physical and cognitive load proper of coordinative exercises (Gallotta et al., 2012, 2015) and therefore, to the less fatiguing cognitive involvement of traditional intervention compared to a mixed physical and cognitive load of coordinative intervention (de Bruijn et al., 2021). However, activities with high coordination demands seemed to be a valid training for executive functions by movement, since the cognitive load required to execute complex movements is crucial for inducing neuroplastic changes that underlie skillful movement performances (Gallotta et al., 2015).

These scientific evidences should be considered when designing physical education school programs in the choice of the contents: indeed, to avoid a potential fatiguing effect on children, especially when the following classrooms are challenging, a balance between coordinative demands and traditional physical exertion could be recommendable.

### 4.3 Relationship between academic achievement variations and several potential predictors

The third aim of this study was to identify potential predictors of children’s academic achievement variations. Results showed that Italian language skill improvement was positively associated with sedentary time and fruit consumption increases. It could be hypothesized that the Italian language study requires more sitting time to be proficiently carried out as they involve writing and thinking tasks. Indeed, as reported in a recent systematic review, school-based physical education programs seem to be more effective on math academic performance than on language and reading abilities (Loturco et al., 2022).

Moreover, although the precise association between fruit and vegetable consumption and academic performance remains uncertain, there are several possible explanations. The nutrients present in fruits and vegetables may play a role in protecting the body against infections and decreasing the likelihood of nutrient deficiencies. Consequently, this could lead to a possible reduction in school absenteeism and allow for more time spent learning (Slavin and Lloyd, 2012; Wolfenden et al., 2021). In addition, vegetables and fruits provide fiber, which helps prevent constipation and consequently may alleviate discomfort and irritability in children, avoiding distractions from learning (Rajindrajith et al., 2013; Wang et al., 2013). Moreover, Bleiweiss-Sande et al. (2019) reported that the consumption of less healthful food in school children was associated with poorer academic achievement, supporting the negative relationship between junk foods and academic achievement. Furthermore, considering that children have smaller stomachs, they often require snacks in addition to meals to ensure sufficient nutrient intake and energy to support their growth and development (Raymond and Morrow, 2022). Therefore, it is possible that incorporating snacks of fruits and vegetables throughout the day might help alleviate hunger and offer a healthy source of energy, potentially enhancing children’s focus on schoolwork or homework.

To the best of our knowledge, no other studies examined the association between such a wide range of health behaviors, physical fitness components, coordinative and cognitive variables and academic achievement among school children. Previous research has addressed mainly to adolescents reporting that frequent consumption of vegetables and fruits, breakfast and dinner with family and regular physical activity were positively associated with higher levels of academic achievement in a 11–15-year-old sample (Faught et al., 2017b). Other results showed that College students adhering to public health recommendations for lifestyle behaviours have modestly higher grades (Wald et al., 2014).

Unexpectedly, our coordinative physical education intervention was associated with a lower probability of improvement in Italian language and mathematical skills. Schmidt et al. (2017) highlighted the positive association between children’s motor coordination and their academic achievement, mediating by executive functions. More recently, de Bruijn et al. (2021) examined the effects of two different 14-week physical education interventions (aerobic versus cognitively-engaging physical activity) on primary school children’s cognition and academic achievement (de Bruijn et al., 2021). They did not find significant differences after interventions between the groups probably due to no significant differences in brain activation changes between the groups, indicating that the type of intervention did not result in changed brain activation patterns and therefore in measurable changes in cognition and academic achievement. These contradictory results could be due to the difficulty to realize physical activity interventions that are purely aerobic or purely coordinative and cognitively-engaging. Moreover, it is also to consider that academic performance is a complex dominion that depends on many interrelated factors such as genetic, socio-economic status, school environment, individual differences and it is also the result of multiple brain functions, such as memory, attention, concentration, making it difficult to interpret conclusions of studies examining the effect of different types of physical activities on children’s executive functions and academic achievement.

The present study is currently being implemented to higher educational stages to investigate if different types of physical activity and sports in combination with healthy lifestyle habits could affect academic achievement in middle-school, high-school, and university students. Specifically, different physical activity and sport interventions could lead to facilitation in the learning process also in adolescents and youngsters in relation to the same abilities reported in the present study or to other academic skills (Gallotta et al., 2020).

## 5 Limitations

Limitations of the study include the lack of assessment of further cognitive functions such as inhibition, shifting, updating and working memory in addition to the attentional performance.

Another limitation is that academic achievement was measured with a scale ranging from 1 to 5 (Henricsson and Rydell, 2006; Thorell et al., 2013). However, this scale was chosen in order to similarly reproduce the Italian primary school grades system which currently ranges from 1 to 4 (corresponding to in the process of first acquisition, base, intermediate, advanced level, respectively and referring to four different learning levels).

It is to note that PAQ-C questionnaire relies on subjective responses from the individual and may not be as accurate or comprehensive as more sophisticated monitoring tools, such as wearable devices that continuously track physical activity.

Finally, the study involved children living in a rural area. Gallotta et al. (2011) verified that living in rural or urban setting can influence training adaptations. Further studies should verify the effect of different physical education interventions on fitness and coordinative abilities in different living settings.

## 6 Conclusion

Motor ability and lifestyle habits may have a positive influence on academic achievement in children. Specifically, aerobic fitness and physical activity level are linked to better academic performance. In the present study, traditional physical education intervention resulted to be more effective on both Italian language and mathematical skills.

Some academic skills are more related to the time spent studying, especially in primary school children who are acquiring some notions for the first time. Therefore, physical education interventions in primary school are necessary to balance and limit the sedentary time.

The complex multicomponent relation between academic achievement and such a wide range of individual variables leads to a large number of interesting discussions and encourages needs to realize further study with rigorous and longitudinal research protocols.

## Data availability statement

The raw data supporting the conclusions of this article will be made available by the authors, without undue reservation.

## Ethics statement

The studies involving humans were approved by The University Ethical Committee of Sapienza University of Rome (Rif 3502 Prot. 1883/15). The studies were conducted in accordance with the local legislation and institutional requirements. Written informed consent for participation in this study was provided by the participants' legal guardians/next of kin.

## Author contributions

MCG: Conceptualization, Data curation, Investigation, Methodology, Writing – original draft. VB: Investigation, Methodology, Writing – review & editing. GZ: Data curation, Formal analysis, Software, Writing – review & editing. DC: Data curation, Formal analysis, Writing – review & editing. LF: Data curation, Investigation, Writing – original draft. SM: Conceptualization, Methodology, Writing – review & editing. LG: Conceptualization, Formal analysis, Methodology, Writing – review & editing. CB: Conceptualization, Methodology, Supervision, Writing – review & editing.
