# Supplementary material for: Academic achievement and healthy lifestyle habits in primary school children: an interventional study
Source: Front Psychol. 2024 Jul 22;15:1412266. doi: 10.3389/fpsyg.2024.1412266 (PMC11298431; doi:10.3389/fpsyg.2024.1412266)
Supplement: Supplementary file 1 [file Table_1.DOCX]

Supplementary Material

# Description of a traditional physical education lesson

# A traditional physical education lesson consisted of a continuous aerobic circuit training followed by a sub-maximal shuttle run exercise. This lesson focused on the improvement of cardiovascular endurance by varying the types of gaits required (e.g., fast walking, running, skipping) without any specific coordinative request. The traditional physical education lesson required changes in executive modalities and some variations of intensity.

# Description of a coordinative physical education lesson

# A coordinative physical education lesson consisted of sport-unspecific use of basketballs in the context of mini-games. The basketballs were used in unconventional ways with varying game rules (e.g., application of foot-eye coordination techniques with basketballs). This lesson was geared toward the development of both motor control and perceptual-motor adaptation abilities. It focused on the development of psychomotor competences and expertise in movement-based problem solving through functional use of a common tool (e.g., basketball), and considering various tasks that involved decision-making motor tasks and manipulative ball handling skills (e.g., bouncing, throwing, and/or receiving a ball). The coordinative physical education lesson combined physical load due to the practice of physical exercises and cognitive load required for movement-based problem solving and decision-making tasks with accurate timing, temporal estimations, temporal production, spatial and temporal adjustments, and spatial and temporal orientation, which are essential cognitive requirements to perform such types of activities. The coordinative physical education lesson aimed to develop both motor control abilities and perceptual-motor adaptation abilities, by combining demands on gross-motor and manipulative control abilities and perceptual-motor adaptation abilities (particularly kinesthetic differentiation and response orientation).
